# Sequential Sinusoidal Obstruction Syndrome and TA-TMA After Haploidentical Hematopoietic Stem Cell Transplantation: Diagnostic and Therapeutic Challenges

**DOI:** 10.3390/jcm15145385

**Published:** 2026-07-09

**Authors:** Gulzhanat Zhunis, Burkitbayev Zhandos, Dina Bashayeva, Vadim Kemaykin, Nazgul Taskhyngali, Mukhambetiyar Karakoz, Aleksandr Kolesnev, Aset Kuttymuratov, Ivan Mishutin, Ruzal Vildanova

**Affiliations:** National Research Oncology Center, Astana 010000, Kazakhstan; guljanat.zhunis@mail.ru (G.Z.); dinabashayeva@gmail.com (D.B.); kemaykin90@mail.ru (V.K.); tasqyngali@gmail.com (N.T.); koles.sasha1966@gmail.com (A.K.); aset91-91@mail.ru (A.K.); ytrewq11101989@gmail.com (I.M.);

**Keywords:** acute myeloid leukemia, haploidentical hematopoietic stem cell transplantation, sinusoidal obstruction syndrome, transplant-associated thrombotic microangiopathy, endothelial dysfunction, multi-organ failure, case report

## Abstract

**Background/Objectives:** Haploidentical hematopoietic stem cell transplantation (haplo-HSCT) has expanded donor availability for patients with acute myeloid leukemia; however, it is associated with a high risk of endothelial complications. Among them, sinusoidal obstruction syndrome (SOS) and transplant-associated thrombotic microangiopathy (TA-TMA) are life-threatening conditions with overlapping clinical features, making early diagnosis and management challenging. **Case Presentation:** We report the case of a 47-year-old woman with acute myeloid leukemia who underwent haploidentical HSCT and developed sequential endothelial complications. On day +11 post-transplant, she presented with weight gain (>5%), hyperbilirubinemia (44 μmol/L), elevated liver enzymes (ALT 2228 U/L and AST 3477 U/L), and multi-organ dysfunction, consistent with probable SOS according to EBMT criteria. Intensive supportive care, glucocorticosteroids, and hemodiafiltration resulted in partial clinical improvement. Subsequently, on day +22, the patient developed progressive thrombocytopenia, hemolytic anemia (hemoglobin 71 g/L), elevated lactate dehydrogenase (3129 U/L), undetectable haptoglobin, and schistocytosis (up to 3.9%), accompanied by neurological symptoms, leading to the diagnosis of TA-TMA. **Results:** Management included discontinuation of tacrolimus, initiation of plasma exchange, and complement inhibition with eculizumab, along with ruxolitinib for graft-versus-host disease prophylaxis. This approach resulted in rapid clinical and laboratory improvement, including resolution of hemolysis, reduction in schistocytes (to 1%), normalization of lactate dehydrogenase, and regression of neurological symptoms. The patient was discharged in stable condition, although requiring ongoing renal replacement therapy. **Conclusions:** This case highlights the complexity of diagnosing and managing sequential endothelial complications after haplo-HSCT. SOS and TA-TMA may reflect overlapping manifestations of endothelial injury rather than entirely isolated complications.

## 1. Introduction

Allogeneic hematopoietic stem cell transplantation is a potentially curative therapeutic procedure for a wide range of malignant and benign hematological diseases. Recent advances in transplantation, including new conditioning regimens and GVHD prophylaxis, have led to significant improvements in the outcomes of hematopoietic stem cell transplantation from alternative donors, particularly in haploidentical hematopoietic stem cell transplantation (haplo-HSCT) [1]. The use of haploidentical donors has increased the availability of this treatment method for patients without a fully compatible donor. However, haplo-HSCT is characterized by a high rate of both infectious and non-infectious complications, including early complications of endothelial origin, the most serious of which are sinusoidal obstruction syndrome (SOS, formerly veno-occlusive liver disease) and transplant-associated thrombotic microangiopathy (TA-TMA) [2,3]. These two conditions have overlapping clinical presentations and can manifest early post-transplant, posing significant diagnostic and therapeutic challenges. Despite advances in haplo-HSCT, early endothelial complications remain poorly understood, particularly in cases of sequential development of SOS and TA-TMA. We present a clinically challenging case of a patient with AML who, following haplo-HSCT, subsequently developed probable SOS with multiple-organ dysfunction and TA-TMA, requiring comprehensive multidisciplinary management.

## 2. Materials and Methods

### 2.1. Study Design

This study represents a descriptive case report conducted in accordance with the CARE guidelines for clinical case reporting.

### 2.2. Patient Information

A 47-year-old female patient with acute myeloid leukemia (AML) was treated at the National Research Oncology Center (Astana, Kazakhstan). Clinical data were retrospectively collected from electronic medical records, including laboratory findings, imaging studies, and treatment interventions.

### 2.3. Transplant Procedure

The patient underwent haploidentical hematopoietic stem cell transplantation (haplo-HSCT) from a related donor. Conditioning regimen included fludarabine and melphalan (Flu-Mel protocol), with graft-versus-host disease (GVHD) prophylaxis consisting of post-transplant cyclophosphamide, tacrolimus, and mycophenolate mofetil.

### 2.4. Diagnostic Criteria

The diagnosis of sinusoidal obstruction syndrome (SOS) was established according to the European Society for Blood and Marrow Transplantation (EBMT) criteria (2024), including weight gain > 5%, hyperbilirubinemia (>34 μmol/L), and clinical signs of organ dysfunction [4]. Transplant-associated thrombotic microangiopathy (TA-TMA) was diagnosed based on international consensus criteria, including the presence of microangiopathic hemolytic anemia (schistocytosis, elevated lactate dehydrogenase, decreased haptoglobin), thrombocytopenia, and organ dysfunction.

### 2.5. Monitoring and Data Collection

The patient was closely monitored through serial laboratory assessments, including complete blood count, liver and renal function tests, lactate dehydrogenase, and hemolysis markers. Imaging studies and echocardiography were performed as clinically indicated.

### 2.6. Therapeutic Interventions

Treatment decisions were made by a multidisciplinary team, including hematologists, intensivists, and hepatologists. Management strategies included immunosuppressive therapy adjustment, glucocorticosteroids, renal replacement therapy, plasma exchange, and targeted therapy with complement inhibition (eculizumab).

## 3. Results

### 3.1. Case Presentation

A 47-year-old female patient with a diagnosis of acute myeloid leukemia (AML) with t(8;21)(q22;q21.3), FAB M2 subtype, and intermediate-risk category according to ELN 2022, achieved complete remission after standard induction chemotherapy (“7 + 3” regimen with daunorubicin 60 mg/m^2^) followed by two cycles of high-dose cytarabine consolidation. Due to the intermediate-risk profile and the availability of a haploidentical related donor (daughter, 19 years old), the patient underwent haploidentical hematopoietic stem cell transplantation (haplo-HSCT). Conditioning regimen included rituximab (375 mg/m^2^, day −8), fludarabine (30 mg/m^2^/day; corresponding absolute daily dose 45 mg, days −7 to −2), and melphalan (70 mg/m^2^/day; corresponding absolute daily dose 110 mg, days −3 and −2). Desensitization with rituximab was performed due to the presence of donor-specific antibodies.

Pre-transplant donor-specific anti-HLA antibodies demonstrated a maximum mean fluorescence intensity (MFI) of 712.93 for class I and 6425.03 for class II antibodies. DSA testing was not repeated after HSCT. Detailed donor–recipient HLA compatibility data are presented in Table 1. Recipient and donor blood groups and red blood cell phenotypes were fully compatible. Both donor and recipient were blood group O, Rh-positive, with a CCDee, Kell-negative phenotype. Anti-erythrocyte antibodies were not detected in the recipient. Peripheral blood stem cells were used as the graft source, with a CD34+ cell dose of 9.3 × 10^6^/kg. Graft-versus-host disease (GVHD) prophylaxis consisted of post-transplant cyclophosphamide (50 mg/kg on days +3 and +4), tacrolimus, and mycophenolate mofetil starting from day +5. Supportive prophylaxis included ursodeoxycholic acid (UDCA) at 12 mg/kg/day starting from the first day of conditioning, sulfamethoxazole/trimethoprim for Pneumocystis jirovecii prophylaxis, acyclovir for antiviral prophylaxis, and fluconazole for antifungal prophylaxis. Baseline ferritin level prior to transplantation was 303.90 μg/L. PCR testing for hepatitis B, hepatitis C, and cytomegalovirus (CMV) was negative before transplantation and during the early post-transplant period. Neutrophil engraftment was achieved on day +12. Donor chimerism reached 98.95% on day +14 and 99.94% on day +29.

### 3.2. Early Post-Transplant Complication: Sinusoidal Obstruction Syndrome

**Figure 1:**
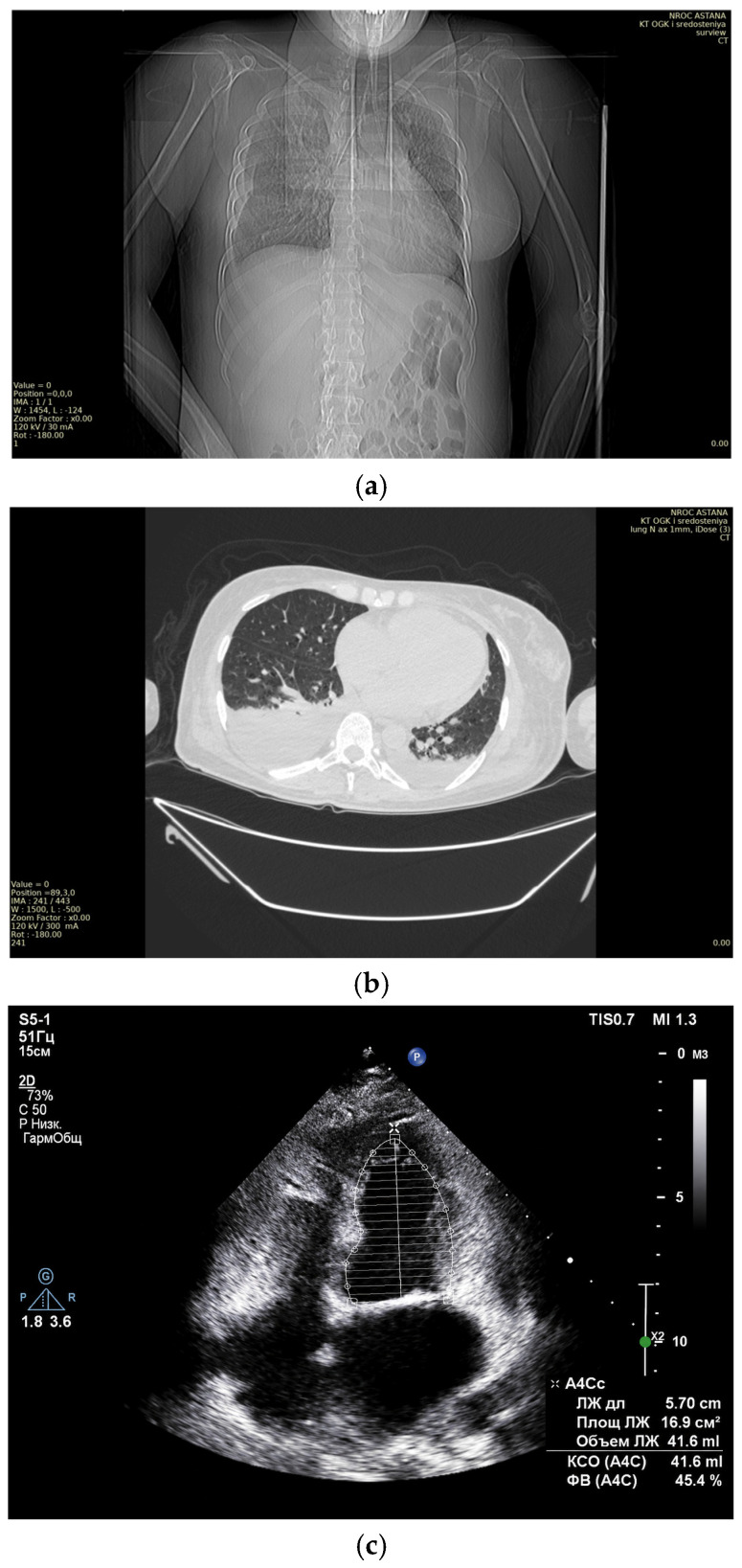
Imaging findings during early post-transplant complications. (**a**) Chest computed tomography demonstrating bilateral pleural effusion and signs of fluid overload. (**b**) Chest X-ray confirming pulmonary congestion. (**c**) Transthoracic echocardiography (apical four-chamber view) showing reduced left ventricular systolic function, with an ejection fraction of 45.4% and a left ventricular volume of 41.6 mL, consistent with cardiac involvement in multi-organ dysfunction.

On day +11, the patient experienced rapid clinical deterioration, including dyspnea (respiratory rate up to 28/min), tachycardia (115–120 bpm), arterial hypertension, oliguria, and weight gain exceeding 5% of baseline. Laboratory findings revealed hyperbilirubinemia (total bilirubin 44 μmol/L, direct bilirubin 25.5 μmol/L), marked elevation of liver enzymes (ALT 2228 U/L and AST 3477 U/L), elevated lactate dehydrogenase (4399 U/L), increased creatinine (136 μmol/L), and coagulopathy (prothrombin index 26.9%, fibrinogen 1.8 g/L). Imaging studies demonstrated right heart dilation, reduced left ventricular ejection fraction (45.4%), mild right-sided hydrothorax, and diffuse hepatic changes (Figure 1).

Note: Imaging findings correspond to the clinical deterioration observed on day +11 after haploidentical hematopoietic stem cell transplantation. Values displayed on the echocardiographic image are presented in the original format.

These findings were considered most compatible with early sinusoidal obstruction syndrome/veno-occlusive disease (SOS/VOD). Hyperbilirubinemia, >5% weight gain, hepatic pain, and the presence of free fluid supported the diagnosis. Liver Doppler ultrasonography did not demonstrate significant portal hypertension or impaired portal blood flow; however, imaging abnormalities may be subtle during the early stages of SOS/VOD. Echocardiography revealed moderately reduced left ventricular systolic function (ejection fraction 42%) without evidence of significant right ventricular overload. The marked elevation of transaminases and LDH was considered compatible with endothelial injury associated with conditioning therapy and GVHD prophylaxis. The absence of fever, low procalcitonin levels, negative blood cultures, and the lack of severe cardiac decompensation or shock made infectious and ischemic causes less likely. The principal differential diagnostic considerations are summarized in Table 2.

According to the 2024 EBMT recommendations, the diagnosis of probable SOS/VOD requires at least two diagnostic criteria, including bilirubin ≥ 2 mg/dL, painful hepatomegaly, weight gain > 5%, ascites, or imaging findings suggestive of SOS/VOD. In the present case, the patient fulfilled three criteria (>5% weight gain, hyperbilirubinemia > 34 μmol/L, and hepatic pain). Considering the timing after haploidentical HSCT, the conditioning regimen, and the overall clinical presentation, a multidisciplinary team involving hematologists, a hepatologist, and an intensivist established the diagnosis of probable SOS/VOD.

Treatment included high-dose glucocorticosteroids (methylprednisolone 2 mg/kg/day), discontinuation of mycophenolate mofetil, renal replacement therapy (hemodiafiltration, total of 8 sessions), inotropic support, and oxygen therapy with escalation to CPAP. Positive changes were noted during therapy by D+22: decreased liver enzymes (ALT 210 U/L, AST 247 U/L), decreased bilirubinemia, and hemodynamic stabilization.

### 3.3. Secondary Complication: Transplant-Associated Thrombotic Microangiopathy

Despite improvement in hepatic function, new clinical and laboratory abnormalities emerged on day +22, including progressive thrombocytopenia (48 × 10^9^/L), anemia (hemoglobin 71 g/L), schistocytosis (up to 3.9%), reticulocytosis (55‰), undetectable haptoglobin (<0.1 g/L), and elevated lactate dehydrogenase (3129 U/L). Neurological symptoms, including confusion and involuntary muscle contractions, were also observed. Based on international diagnostic criteria, transplant-associated thrombotic microangiopathy (TA-TMA) was diagnosed. The dynamic evolution of hematologic, renal, and hemolysis-related parameters supporting the diagnosis of TA-TMA is summarized in Table 3. Management included discontinuation of tacrolimus, initiation of plasma exchange (two sessions), and targeted therapy with eculizumab (900 mg weekly for two doses followed by 300 mg). GVHD prophylaxis was modified to ruxolitinib (10 mg/day). This therapeutic approach resulted in significant clinical and laboratory improvement, including reduction in schistocytes to 1% by day +36, normalization of hemolysis parameters, and resolution of neurological symptoms.

According to the “Concordant Diagnostic Criteria for TA-TMA” proposed by the Expert Harmonization Group, the diagnosis of TA-TMA requires fulfillment of at least 4 of 7 diagnostic criteria at two consecutive time points over a 14-day period. Although the laboratory findings in this case did not fully satisfy all harmonized criteria, the patient fulfilled 4 of the 7 diagnostic criteria, including transfusion-dependent cytopenias (thrombocytopenia and a hemoglobin decrease ≥ 10 g/L from baseline), elevated LDH, and schistocytosis on peripheral blood smear. In addition, several recognized risk factors for TA-TMA were present, including melphalan-based conditioning, haploidentical HSCT, post-transplant cyclophosphamide, and calcineurin inhibitor exposure for GVHD prophylaxis.

Based on the combination of clinical manifestations, laboratory abnormalities, progressive renal dysfunction, neurological symptoms, and dynamic clinical evolution, transplant-associated thrombotic microangiopathy (TA-TMA) was diagnosed. The diagnostic process was further complicated by limited access to complement-specific biomarkers and exclusion tests, including sC5b-9, anti-CFH antibodies, and ADAMTS13 activity.

Currently, TA-TMA is stratified into standard-risk and high-risk forms. According to contemporary criteria, patients presenting with at least one high-risk feature, including peak LDH ≥ 2× the upper limit of normal, rUPCR ≥ 1 mg/mg, TMA-associated organ dysfunction (excluding KDIGO stage I acute kidney injury), elevated soluble C5b-9 levels, concomitant grade II–IV acute graft-versus-host disease, or systemic infection—should be considered for targeted therapy [5]. In the present case, the patient fulfilled two high-risk criteria, including marked elevation of LDH and significant organ dysfunction. In addition, several recognized risk factors for TA-TMA were present, including melphalan-based conditioning, haploidentical HSCT, post-transplant cyclophosphamide, and calcineurin inhibitor exposure for GVHD prophylaxis. These findings supported the diagnosis of high-risk TA-TMA and the need for targeted treatment. However, due to the temporary unavailability of the complement inhibitor eculizumab at the time of diagnosis, treatment was initially started with two sessions of plasma exchange on days +22 and +23. Subsequently, eculizumab was administered intravenously at a dose of 900 mg. In total, the patient received two weekly doses of 900 mg followed by one weekly dose of 300 mg.

Immunosuppressive therapy was modified in accordance with the TA-TMA treatment protocol. Tacrolimus was discontinued, and graft-versus-host disease prophylaxis was transitioned to oral ruxolitinib at a dose of 10 mg/day divided into two doses. Supportive therapy and renal replacement therapy were continued due to persistent anuria and severe acute kidney injury corresponding to RIFLE stage F.

Following combination therapy with eculizumab and ruxolitinib, marked clinical and laboratory improvement was observed. By day +36, the percentage of schistocytes in peripheral blood decreased from 3.9% to 1.0%, accompanied by a significant reduction in LDH levels. Hemolysis resolved, eliminating the need for further red blood cell transfusions, and neurological manifestations gradually regressed.

### 3.4. Outcome

By days +36 to +40, the patient’s condition stabilized, with recovery of hepatic and respiratory function. However, renal impairment persisted, requiring ongoing hemodialysis. The patient was discharged on day +40 in stable condition (ECOG 1–2) for outpatient follow-up and continuation of renal replacement therapy. A chronological summary of the patient’s clinical course, therapeutic interventions, and sequential endothelial complications is presented in Table 4.

## 4. Discussion

The present case highlights the complexity of managing early endothelial complications following haploidentical hematopoietic stem cell transplantation (haplo-HSCT), particularly when they occur sequentially in a single patient. Sinusoidal obstruction syndrome (SOS) and transplant-associated thrombotic microangiopathy (TA-TMA) are both well-recognized but potentially life-threatening complications, sharing overlapping clinical features and common underlying mechanisms related to endothelial injury [4,6].

The sequential development of SOS/VOD and TA-TMA in this patient highlights the clinical and biological overlap between post-transplant endothelial complications [7,8]. Although current evidence suggests that both syndromes are associated with endothelial injury, the concept of a unified “endothelial continuum” remains hypothetical. This clinical observation does not establish a direct causal relationship between SOS/VOD and TA-TMA; however, it may reflect the sequential manifestation of overlapping endothelial dysfunction following haplo-HSCT in a susceptible patient. Conditioning regimens, calcineurin inhibitors, inflammatory cytokine release, and alloimmune reactions all contribute to endothelial injury, which may evolve dynamically over time [9,10].

One of the key challenges illustrated by this case is the difficulty of differential diagnosis in the early post-transplant period. Differential diagnosis between these conditions and other causes of multi-organ failure, such as sepsis, drug-induced toxicity, or graft-versus-host disease, remains highly challenging, particularly in cases of sequential or overlapping development [7,8,9,10]. SOS is typically characterized by weight gain, hyperbilirubinemia, and hepatic dysfunction, whereas TA-TMA presents with microangiopathic hemolytic anemia, thrombocytopenia, and organ dysfunction [4,10].

Although the clinical presentation was considered most consistent with probable SOS/VOD according to EBMT criteria, the diagnosis could not be established with complete certainty. The patient fulfilled three diagnostic criteria (>5% weight gain, hyperbilirubinemia, and hepatic pain); however, clinically significant hepatomegaly and definitive Doppler ultrasound signs of portal hypertension were absent. In addition, current EBMT recommendations emphasize the importance of excluding alternative causes of liver dysfunction. Extensive differential diagnostic evaluation was therefore performed, including assessment for sepsis-associated liver injury, drug-induced liver injury, ischemic liver injury, cardiac dysfunction-associated liver injury, and early TA-TMA. While these alternative diagnoses were considered less likely based on the overall clinical presentation and treatment response, they could not be completely excluded.

Importantly, this case underscores the need for continuous reassessment and dynamic monitoring of laboratory and clinical parameters. Early identification of schistocytes, elevated lactate dehydrogenase, and decreased haptoglobin was critical in establishing the diagnosis of TA-TMA [10]. These findings highlight the importance of incorporating routine screening for hemolysis markers in high-risk patients following haplo-HSCT.

From a therapeutic perspective, the management of endothelial complications remains challenging and requires a multidisciplinary approach. In the absence of defibrotide, which is considered the standard of care for probable SOS, alternative strategies such as glucocorticosteroids and intensive supportive therapy may be applied, although evidence remains limited [5].

Complement activation is increasingly recognized as a central mechanism in the pathogenesis of TA-TMA. In this context, complement inhibition with eculizumab has emerged as a targeted and effective treatment option, particularly in severe cases with organ involvement [11,12,13,14,15,16,17,18,19,20,21]. In our patient, initiation of eculizumab therapy, combined with withdrawal of tacrolimus and transition to ruxolitinib, resulted in rapid resolution of hemolysis and neurological symptoms, supporting its clinical efficacy.

Notably, while plasma exchange was initiated as a bridging therapy, its role in transplant-associated TMA remains limited, and current evidence suggests that it should not delay the initiation of complement-targeted treatment [12,13,14,15,16,17,18]. This case further reinforces the importance of early access to targeted therapies in improving outcomes.

Another important clinical implication of this report is the role of immunosuppressive management. Calcineurin inhibitors are a known risk factor for endothelial toxicity and TA-TMA, and their timely discontinuation is crucial. The successful use of ruxolitinib in our case highlights its potential dual benefit in GVHD prophylaxis and modulation of inflammatory pathways contributing to endothelial injury [13,14,15,16].

This case has several limitations inherent to single case reports, including limited generalizability. However, it provides valuable clinical insight into the dynamic nature of endothelial complications and emphasizes the importance of early recognition, differential diagnosis, and individualized therapeutic strategies. The absence of complement-specific biomarkers, including soluble C5b-9, limited the ability to further characterize complement activation in this patient.

This case demonstrates that SOS and TA-TMA may occur sequentially after haplo-HSCT and may represent overlapping manifestations of endothelial dysfunction.

## 5. Conclusions

Haploidentical hematopoietic stem cell transplantation remains a complex but often the only curative option for patients with acute myeloid leukemia. This case demonstrates that SOS and TA-TMA may occur sequentially after haplo-HSCT and may present with overlapping manifestations of endothelial dysfunction.

Early recognition, continuous reassessment, and prompt differentiation between these conditions are critical, as therapeutic strategies differ significantly. Timely modification of immunosuppressive therapy and initiation of targeted treatment, particularly complement inhibition, may improve clinical outcomes even in the setting of multi-organ dysfunction.

This report emphasizes the importance of multidisciplinary management, routine monitoring of hemolysis markers, and heightened clinical vigilance for evolving endothelial complications in patients undergoing haplo-HSCT.

## Figures and Tables

**Table 1:** Donor and recipient HLA genotypes determined by next-generation sequencing (NGS).

Donor HLA Genotype by NGS (Female, 19 Years Old)		
Locus	Allele 1	Allele 2
A	01:01	24:02
B	40:02	58:01
C	03:02	03:04
DRB1	03:01	04:01
DQB1	02:01	03:01
**Recipient HLA Genotype by NGS (Female, 47 Years Old)**		
Locus	Allele 1	Allele 2
A	02:10 *	24:02
B	40:02	40:06 *
C	03:04	08:01 *
DRB1	04:01	12:01 *
DQB1	03:01 *	03:01

* Loci for which the recipient had no matches with the donor were noted.

**Table 2:** Differential diagnostic considerations during early post-transplant multiorgan dysfunction.

Condition	Arguments “FOR”	Arguments “AGAINST”
SOS/VOD	• Day +11 after haplo-HSCT (typical timing) • Weight gain > 5% • Bilirubin 44 µmol/L • Hepatic pain • Free fluid in the pelvis • Very high AST/ALT and LDH • Tacrolimus level 17 ng/mL • Possible minimal ultrasound signs at an early stage • Signs of venous endothelial injury • Risk factors: haploidentical HSCT, melphalan-based conditioning regimen, GVHD prophylaxis with post-transplant cyclophosphamide (PTCy)	• No pronounced hepatomegaly • No massive ascites • No signs of reversed portal venous flow • Portal vein not dilated
Drug toxicity (drug-induced liver injury)	• Possible toxicity related to conditioning regimen and GVHD prophylaxis • Elevated tacrolimus level, although not critically high (less typical for such marked cytolysis)	• Does not fully explain significant weight gain • Hyperbilirubinemia accompanied by pronounced hemolysis
Sepsis-associated liver injury	• CRP 76 elevated, though not critically. Early after HSCT, CRP elevation may occur even in the setting of mucositis	• Procalcitonin normal (0.2 ng/mL) • No fever • Blood cultures negative • No clinical signs of sepsis or septic shock • No obvious infectious focus
TA-TMA	• Very high LDH 4399 • Rising creatinine to 136 µmol/L • Elevated tacrolimus level • Possible overlap with SOS/VOD	• No schistocytes on peripheral blood smear at that stage • No newly developed or refractory thrombocytopenia and/or progressive anemia • No proteinuria • No refractory arterial hypertension
Ischemic liver injury (shock liver)	• AST/ALT > 2000–3000 • Very high LDH • Possible impaired perfusion	• No documented hypotension/shock • No severe cardiac decompensation • Echocardiography does not explain such severe cytolysis

**Table 3:** Dynamic clinical and laboratory findings supporting the diagnosis of TA-TMA.

Parameter	Baseline Level (Before Suspected TA-TMA)	Level at Time of Differential Diagnosis/Suspected TA-TMA	Maximum/Minimum Level	After Therapy
Anemia/Thrombocytopenia	Hb 89 g/L PLT 14 × 10^9^/L	Hb 71 g/L PLT 48 × 10^9^/L	Hb 64 g/L PLT 11 × 10^9^/L	Hb 71 g/L PLT 20–30 × 10^9^/L without transfusions
LDH/Schistocytes	1492 U/L Schistocytes not assessed	3129 U/L 33 per 1000 erythrocytes	3129 U/L 39 per 1000 erythrocytes	702 U/L 10 per 1000 erythrocytes
sC5b-9 level	Not assessed	Not assessed	Not assessed	Not assessed
rUPCR (urine protein-to-creatinine ratio)	Urine protein 210 mg/L Urine creatinine 497.7 mg/L rUPCR 0.4 mg/mg	Urine protein 3000 mg/L Urine creatinine 2000 mg/L rUPCR 1.5 mg/mg	Urine protein 3200 mg/L Urine creatinine 497 mg/L rUPCR 6.4 mg/mg	Not assessed
Haptoglobin	Not assessed	0 g/L	Not assessed	Not assessed
DAT (Direct Antiglobulin Test)	Negative	Negative	Negative	Negative
Blood tacrolimus level	18.30 ng/mL	15.57 ng/mL	2.2 ng/mL after tacrolimus discontinuation	Not assessed
Creatinine/Urea	Creatinine 135 µmol/L Urea 13 mmol/L	Creatinine 270 µmol/L Urea 20 mmol/L	Creatinine 373 µmol/L Urea 20 mmol/L	Creatinine 281 µmol/L Urea 10 mmol/L
Neurological symptoms	Absent	Mixed encephalopathy due to decompensated liver failure. Glasgow Coma Scale 13, contact difficult, reduced insight, incomplete orientation in place and time. Upper limb weakness up to 3.5 points.	Not assessed	Completely resolved
ALT	2356.14 U/L	210 U/L	90 U/L	70 U/L
AST	2749.09 U/L	247 U/L	98 U/L	92 U/L
Total bilirubin	92 µmol/L	57 µmol/L	Not assessed	14 µmol/L
Direct bilirubin	72 µmol/L	29 µmol/L	Not assessed	7.6 µmol/L

**Table 4:** Summary of the clinical course, highlighting the sequential development of SOS and TA-TMA after haplo-HSCT.

Day	Clinical Event
D-8	Desensitization: Rituximab 600 mg IV
D-7-D-2	Conditioning (Flu-Mel): fludarabine 45 mg/day (equivalent to 30 mg/m^2^/day), melphalan 110 mg/day (equivalent to 70 mg/m^2^/day)
D0	Haploidentical HSCT (peripheral blood stem cells, CD34+ 9.3 × 10^6^/kg)
D+1	Grade 1 cytokine release syndrome (fever, IL-6 elevation), resolved with glucocorticosteroids
D+3, D+4	GVHD prophylaxis: cyclophosphamide 50 mg/kg/day; mycophenolate mofetil 30 mg/kg/day orally.
D+5	Initiation of tacrolimus and mycophenolate mofetil
D+8	Grade 1–2 mucositis, transition to IV therapy
D+11	Clinical deterioration: suspected probable SOS (weight gain > 5%, hyperbilirubinemia, elevated ALT, AKI). ICU transfer
D+12	Neutrophil engraftment. Cardiovascular support initiated. Hemodiafiltration started
D+14	Donor chimerism: 98.95%
D+22	Diagnosis of TA-TMA: anemia, thrombocytopenia, schistocytes (3.3%), LDH increase, neurological symptoms, renal failure
D+22-D+23	Plasma exchange therapy initiated. Tacrolimus discontinued
D+23	Eculizumab initiated (900 mg). Immunosuppression switched to ruxolitinib.
D+29	Improvement: decrease LDH, decrease of schistocytes (to 1%), neurological recovery, donor chimerism 99.94%
D+36-D+40	Stabilization; persistent renal dysfunction requiring hemodialysis
D+40	Discharge (ECOG 1–2), outpatient follow-up

## Data Availability

The original contributions presented in this study are included in the article. Further inquiries can be directed to the corresponding authors.

## Abbreviations

The following abbreviations are used in this manuscript:

AML	Acute Myeloid Leukemia
HSCT	Hematopoietic Stem Cell Transplantation
haplo-HSCT	Haploidentical Hematopoietic Stem Cell Transplantation
SOS	Sinusoidal Obstruction Syndrome
TA-TMA	Transplant-Associated Thrombotic Microangiopathy
GVHD	Graft-Versus-Host Disease
LDH	Lactate Dehydrogenase
ALT/AST	Alanine Aminotransferase/Aspartate Aminotransferase
EF	Ejection Fraction
EBMT	European Society for Blood and Marrow Transplantation
ELN 2022	European LeukemiaNet 2022
CPAP	Continuous positive airway pressure
